# *NTRK* Fusion Genes in Thyroid Carcinomas: Clinicopathological Characteristics and Their Impacts on Prognosis

**DOI:** 10.3390/cancers13081932

**Published:** 2021-04-16

**Authors:** Barbora Pekova, Vlasta Sykorova, Karolina Mastnikova, Eliska Vaclavikova, Jitka Moravcova, Petr Vlcek, Petr Lastuvka, Milos Taudy, Rami Katra, Petr Bavor, Daniela Kodetova, Martin Chovanec, Jana Drozenova, Jaromir Astl, Petr Hrabal, Josef Vcelak, Bela Bendlova

**Affiliations:** 1 Department of Molecular Endocrinology, Institute of Endocrinology, 11694 Prague, Czech Republic; vsykorova@endo.cz (V.S.); kmastnikova@endo.cz (K.M.); evaclavikova@endo.cz (E.V.); jmoravcova@endo.cz (J.M.); jvcelak@endo.cz (J.V.); bbendlova@endo.cz (B.B.); 2 Department of Nuclear Medicine and Endocrinology, 2nd Faculty of Medicine, Charles University and Motol University Hospital, 15006 Prague, Czech Republic; petr.vlcek@fnmotol.cz; 3 Department of Otorhinolaryngology and Head and Neck Surgery, 1st Faculty of Medicine, Charles University and Motol University Hospital, 15006 Prague, Czech Republic; petr.lastuvka@fnmotol.cz (P.L.); milos.taudy@fnmotol.cz (M.T.); 4 Department of Ear, Nose and Throat, 2nd Faculty of Medicine, Charles University and Motol University Hospital, 15006 Prague, Czech Republic; rami.katra@fnmotol.cz; 5 Department of Surgery, 2nd Faculty of Medicine, Charles University and Motol University Hospital, 15006 Prague, Czech Republic; petr.bavor@fnmotol.cz; 6 Department of Pathology and Molecular Medicine, 2nd Faculty of Medicine, Charles University and Motol University Hospital, 15006 Prague, Czech Republic; daniela.kodetova@fnmotol.cz; 7 Department of Otorhinolaryngology, Charles University, 3rd Faculty of Medicine, University Hospital Kralovske Vinohrady, 10034 Prague, Czech Republic; martin.chovanec@fnkv.cz; 8 Department of Pathology, Charles University, 3rd Faculty of Medicine, University Hospital Kralovske Vinohrady, 10034 Prague, Czech Republic; jana.drozenova@fnkv.cz; 9 Department of Otorhinolaryngology and Maxillofacial Surgery, Military University Hospital, 16902 Prague, Czech Republic; jaromir.astl@uvn.cz; 10 Department of Pathology, Military University Hospital, 16902 Prague, Czech Republic; petr.hrabal@uvn.cz

**Keywords:** *NTRK*, fusion gene, papillary thyroid carcinoma, poorly differentiated thyroid carcinoma, clinicopathological feature, follow-up, prognosis, outcome

## Abstract

**Simple Summary:**

*NTRK* fusion genes are important but not well studied molecular markers in thyroid cancer. Their identification could help improve diagnosis and prognosis, and determine appropriate treatment. The aims of this study were to identify *NTRK* fusion-positive thyroid tumors in a large cohort of different thyroid tumors, to characterize these tumors by molecular, clinical and pathological features and to evaluate the impact of *NTRK*-rearranged tumors on prognosis of the disease. A suitable approach for selective *NTRK* fusion gene testing in thyroid cancer samples was created and utilized. In a cohort of 59 *NTRK*-rearranged carcinomas, characteristic features were described and recommendations for surgery and prognostic factors were determined thanks to the long-term follow-up of patients.

**Abstract:**

Chromosomal rearrangements of *NTRK* genes are oncogenic driver mutations in thyroid cancer (TC). This study aimed to identify *NTRK* fusion-positive thyroid tumors and to correlate them with clinical and pathological data and determine their prognostic significance. The cohort consisted of 989 different TC samples. Based on the detected mutation, samples were triaged, and those that were positive for a *BRAF*, *HRAS*, *KRAS*, *NRAS, RET*, *RET/PTC* or *PAX8/PPARγ* mutation were excluded from further analyses. *NTRK* fusion gene testing was performed in 259 cases, including 126 cases using next-generation sequencing. *NTRK* fusion genes were detected in 57 of 846 (6.7%) papillary thyroid carcinomas and in 2 of 10 (20.0%) poorly differentiated thyroid carcinomas. A total of eight types of *NTRK* fusions were found, including *ETV6/NTRK3*, *EML4/NTRK3*, *RBPMS/NTRK3*, *SQSTM1/NTRK3*, *TPM3/NTRK1*, *IRF2BP2/NTRK1*, *SQSTM1/NTRK1* and *TPR/NTRK1.* *NTRK* fusion-positive carcinomas were associated with the follicular growth pattern, chronic lymphocytic thyroiditis and lymph node metastases. *NTRK1*-rearranged carcinomas showed a higher frequency of multifocality and aggressivity than *NTRK3*-rearranged carcinomas. Tumor size, presence of metastases, positivity for the *NTRK3* or *NTRK1* fusion gene and a late mutation event (*TERT* or *TP53* mutation) were determined as factors affecting patient prognosis. *NTRK* fusion genes are valuable diagnostic and prognostic markers.

## 1. Introduction

Thyroid cancer (TC) is the most common endocrine malignancy with a still increasing incidence. The prognosis of TC varies primarily depending on the type and stage of the tumor. In the case of papillary thyroid carcinoma (PTC), the prognosis is very favorable and the 10-year overall survival rate is 97% [1]. On the other hand, poorly differentiated thyroid carcinoma (PDTC) carries a worse prognosis, and anaplastic thyroid carcinoma (ATC) has a median survival of 6 months [2]. The clinical outcome of the disease is associated with various mutations occurring in TC. The most common mutation in TC is the *BRAF* V600E mutation, which has appeared to be associated with a higher risk of cancer recurrence [3]. However, this association has recently been disputed [4]. *TERT* promoter mutations are associated with distant metastases and a higher risk of mortality in advanced cases of TC [5]. Differentiated thyroid tumors harboring *RAS* mutations without any co-alterations have excellent prognosis [6]. Other genetic alterations occurring in TC are rearrangements of *NTRK*, *RET*, *ALK*, *BRAF*, *MET*, *FGFR*, *PPARγ* or *ROS1* genes whose associations with the outcome of the disease are not yet well known.

A rearrangement involving one of the neurotrophic-tropomyosin receptor kinase (*NTRK*) genes belonging to the *NTRK* family represents a significant oncogenic event in TC. The *NTRK* family includes three genes, *NTRK1*, *NTRK2* and *NTRK3*, encoding tropomyosin receptor kinases A, B and C, respectively. Fusions involving a kinase domain of *NTRK* gene lead to uncontrolled activation of the tyrosine receptor kinase (TRK) and subsequently the MAPK, PI3K/AKT and PLCγ pathways [7].

In TC, *NTRK* fusions have been reported in PTC, Hürthle cell carcinoma (HCC), PDTC and ATC [8,9]. The presence of *NTRK* fusions in TC is rare and the frequency ranges from 2.3 to 3.4% in predominantly adult cohorts [8,10,11,12]. In pediatric TC, *NTRK* fusions are approximately eight times more common than in adults; the frequency is between 18.3% and 25.9% [13,14,15]. Clinicopathological characteristics and long-term disease development are not well understood due to limited *NTRK* fusion-positive cohorts. Recently, there has been a growing interest in testing and characterizing *NTRK* fusion genes because they are therapeutically targetable. *NTRK* fusion-positive tumors are sensitive to TRK inhibitors, such as larotrectinib, which appears to be well tolerated and effective [16].

This study aimed to determine clinical and pathological features of a large cohort of *NTRK* fusion-positive thyroid tumors, to compare these characteristics between *NTRK3* and *NTRK1* fusion-positive tumors and to determine the prognostic significance of *NTRK* fusion genes based on long-term follow-up of patients with TC harboring this mutation.

## 2. Materials and Methods

### 2.1. The Cohort

A total of 989 (851 PTCs, 86 medullary thyroid carcinomas (MTCs), 19 follicular thyroid carcinomas (FTCs), 13 ATCs, 10 PDTCs and 10 HCCs) thyroid cancer tissues, 30 borderline thyroid tumor tissues (17 follicular tumors of uncertain malignant potential (FT-UMPs), 13 non-invasive follicular tumors with papillary-like nuclear features (NIFTPs)) and 194 benign thyroid tissues were collected from patients who underwent thyroid surgery at the Motol University Hospital (between years 2003 and 2020), the University Hospital Kralovske Vinohrady (between years 2016 and 2020) and the Military University Hospital (between years 2019 and 2020) in Prague. All samples except 65 formalin-fixed paraffin-embedded (FFPE) were fresh frozen thyroid tissues. Detailed clinical and pathological data were collected from all patients whose tumors were positive for the NTRK fusion gene. 

### 2.2. Nucleic Acid Extraction

DNA and RNA were extracted from fresh frozen thyroid tissues using the AllPrep DNA/RNA/Protein Mini Kit (Qiagen, Venlo, Netherlands) or the AllPrep DNA/RNA/miRNA Universal Kit (Qiagen) and from FFPE thyroid tissues using the AllPrep DNA/RNA FFPE Kit (Qiagen) according to the instructions in the manuals. The concentrations and purity of samples were measured using a fluorometer (Qubit 2.0, Invitrogen, Carlsbad, CA, USA) and a spectrophotometer (QIAxpert, Qiagen), respectively.

### 2.3. NTRK Fusion Genes in Pediatric Samples

Analyses of *NTRK* fusion genes in pediatric PTC specimens (*n* = 93) using the QIASeq Targeted RNAscan panel (Qiagen) or the FusionPlex Comprehensive Thyroid and Lung (CTL) panel (ArcherDx, Boulder, CO, USA) were described previously [13]. New pediatric PTC samples collected during year 2020 (*n* = 11) were analyzed by the same procedure as the adult TC samples described below.

### 2.4. Analyses of Point Mutations, RET/PTC and PAX8/PPARγ Rearrangements

First, detection of mutations in the *BRAF*, *HRAS*, *KRAS*, *NRAS*, *TERT*, and *TP53* genes, and *RET/PTC1, RET/PTC3*, and *PAX8/PPARγ* rearrangements in PTC, ATC, PDTC, FTC and HCC samples was performed. Mutations in the *RET*, *HRAS* and *KRAS* genes were analyzed in MTC samples. Libraries were prepared from purified PCR products of genes *BRAF* (exon 15), *HRAS* (exons 2 and 3), *KRAS* (exons 2 and 3), *NRAS* (exons 2 and 3), *TP53* (exons 4, 5, 6, 7, 8 and 9) and *RET* (exons 8, 10, 11, 13, 14, 15 and 16) using the Nextera XT DNA Library Prep Kit (Illumina, San Diego, CA, USA) and subsequently sequenced on the MiSeq sequencer (Illumina). The promoter of the *TERT* gene was screened for the C228T and C250T mutations using capillary sequencing on the CEQ 8000 instrument (Beckman Coulter, Brea, CA, USA). A more detailed procedure was described in our previous study [17]. 

For *RET/PTC* and *PAX8/PPARγ* rearrangements detection, total RNA was reverse transcribed into cDNA by the same procedure as reported previously [13]. Subsequently, cDNA was diluted five times and amplified using the TaqMan Fast Advanced Master Mix (Applied Biosystems, Foster City, CA, USA), gene-specific primers and hydrolysis probes designed using the Primer3Plus software tool. Primer and probe sequences are summarized in Supplementary Table S1. Amplification of the *ACTB* gene was used as a cDNA quality control. Each experiment included a negative control where RNase-free water was used instead of template cDNA. Real-time PCR was performed as follows by the Light Cycler^®^ 480 (Roche, Penzberg, Germany): 50 °C for 2 min, 95 °C for 20 s, followed by 40 cycles of 95 °C for 3 s and 60 °C for 30 s. The evaluation was performed by the Light Cycler^®^ 480 SW 1.5.1. (Roche).

### 2.5. NTRK Fusion Genes Detection

Based on the previously detected mutations, specimens were selected for analyses of the *NTRK* fusion genes. Samples positive for the *BRAF*, *HRAS*, *KRAS*, *NRAS, RET*, *RET/PTC* or *PAX8/PPARγ* mutation were excluded from the further analyses. In contrast, samples in which the *TERT* or *TP53* mutation was identified were included in further testing.

A total of 259 (205 PTCs, 16 sporadic MTCs, 13 FTCs, 9 ATCs, 6 PDTCs, and 10 HCCs) thyroid tissue samples were retrospectively analyzed using the *NTRK* Gene Fusions Detection Kit (AmoyDx, Xiamen, China) according to the manufacturer’s instructions. This Real-Time PCR kit allows to detect 109 different *NTRK1*, *NTRK2* or *NTRK3* fusions. Real-time PCR was performed using the Light Cycler^®^ 480 (Roche) and the results were evaluated by the Light Cycler^®^ 480 SW 1.5.1. (Roche). 

An extended analysis of next-generation sequencing was performed for a total of 126/259 thyroid tissue samples (93 PTCs, 16 sporadic MTCs, 2 FTCs, 9 ATCs, and 6 PDTCs) using the FusionPlex CTL panel (ArcherDx) allowing to detect novel fusion genes as well. The manufacturer’s instructions were followed. Briefly, RNA was reverse transcribed using random primers into cDNA. The adapters containing unique molecular tags and sample indexes (i5) were ligated to modified ends of cDNA. Then, two rounds of PCR followed. Other sample indexes (i7) were added during the second PCR. Purified and quantified libraries were paired-end sequenced on the MiSeq sequencer (Illumina). Bioinformatic analysis was performed using the Archer Analysis software version 6.0.4. (ArcherDx). For a gene fusion to be considered as valid, at least five high-quality unique reads had to span the breakpoint and a minimum of three reads had to have a unique start site.

The gene-specific primers and hydrolysis probes were designed for all identified *NTRK* fusion genes using the Primer3Plus software tool. Primer and probe sequences are summarized in Supplementary Table S1. All *NTRK* fusion-positive samples were verified using these primers and probes by real-time PCR. PCR conditions were the same as for *RET/PTC* or *PAX8/PPARγ* rearrangements detection. A total of 30 borderline thyroid samples and 194 benign thyroid samples were screened by this procedure for all these identified *NTRK* fusion genes.

### 2.6. Statistical Analysis

Categorical variables were compared by use of the Fisher’s exact test, and continuous variables were compared by use of the t-test. Statistical analyses were performed using the Simple Interactive Statistical Analysis (SISA, https://www.quantitativeskills.com/sisa/) and GraphPad tools (GraphPad Software, San Diego, CA, USA). A *p* value < 0.05 was considered as statistically significant.

## 3. Results

### 3.1. Clinical and Pathological Features

The analyses revealed *NTRK* fusion gene in 59 patients with TC, in 57 of 846 (6.7%) patients with PTC in 2 of 10 (20.0%) patients with PDTC. No *NTRK* fusion gene was detected in 16 MTCs, 13 FTCs, 9 ATCs, 10 HCCs, 30 borderline thyroid tumors or 190 benign thyroid tissues subjected to *NTRK* fusion analyses. cDNA from five FFPE PTCs and four benign fresh frozen thyroid tissues were not of sufficient quality for analyses.

Clinical and pathological data of patients with TC harboring *NTRK* fusion are summarized in Table 1. In this cohort, the female to male ratio was 3.5:1 and the mean age of diagnosis was 32.8 ± 16.0 (range 6–77 years). The cohort consisted of 17 pediatric (˂20 years of age) and of 42 adult patients. Before the diagnosis of TC, only one patient suffered from a malignant disease (sigmoid colon cancer) and underwent radiation therapy. Four patients underwent hemithyroidectomy followed by total thyroidectomy, 55 patients underwent total thyroidectomy during one surgery and 37 patients also underwent lymph node (LN) dissection. The mean tumor size was 20.9 ± 11.9 mm (range 5–55 mm). A microcarcinoma (≤10 mm) was revealed in 13.6% of patients. A follicular growth pattern was shown to be present in 78.0% of cases, of which in 47.5% of cases it formed the whole tumor or was predominant, and in 30.5% of cases it was found mixed with a papillary growth pattern (approximately in a similar proportion). Only 13.6% of cases had predominantly papillary architecture. Both cases of PDTC had coexisting PTC component with a mixed papillary and follicular growth pattern. Multifocality, extrathyroidal extension and intravascular invasion were identified in 44.1%, 40.7% and 23.5% of cases, respectively. LN metastases were found in 54.2% of patients and distant metastases in 6.8% of patients, in all cases affecting lungs. Chronic lymphocytic thyroiditis (CLT) was identified in 65.5% of cases. Detailed data on individual cases are described in Supplementary Table S2.

### 3.2. NTRK Fusion Genes

**Figure 1:**
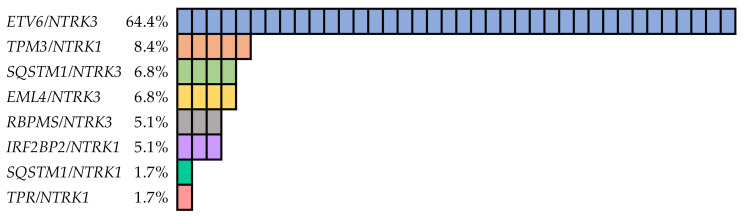
Frequencies of different types of *NTRK* fusion genes detected in thyroid cancer.

A total of eight types of *NTRK* fusions were found, including *ETV6/NTRK3* (four isoforms), *EML4/NTRK3*, *RBPMS/NTRK3*, *SQSTM1/NTRK3*, *TPM3/NTRK1*, *IRF2BP2/NTRK1* (two isoforms), *SQSTM1/NTRK1* and *TPR/NTRK1* (Figure 1). All fusion genes were in-frame. Transcripts of genes involved are listed in Supplementary Table S3. The *ETV6/NTRK3* fusion gene represented the majority (38/59) of positive cases. The most common isoform was the *ETV6* (exon 4)/*NTRK3* (exon 14), which was detected in one case together with the *ETV6* (exon 5)/*NTRK3* (exon 14) isoform. Furthermore, in one PDTC case, two rare isoforms *ETV6* (exon 2)/*NTRK3* (exon 14) and *ETV6* (exon 5)/*NTRK3* (exon 15) were identified. The *TPM3/NTRK1* fusion gene was detected in a significantly smaller group of samples (5/59), similarly to the *SQSTM1/NTRK3* (4/59), *EML4/NTRK3* (4/59), *RBPMS/NTRK3* (3/59) and *IRF2BP2/NTRK1* (3/59) fusion genes. Two isoforms of the *IRF2BP2/NTRK1* fusion gene, *IRF2BP2* (exon 1)*/NTRK1* (exon 10) in two cases and *IRF2BP2* (exon 2)*/NTRK1* (exon 10) in one case were identified. The *SQSTM1/NTRK1* was identified in the second PDTC case and the *TPR/NTRK1* was found in one PTC.

Co-alterations were identified in two samples and in both cases with *TPM3/NTRK1*. In the first case, the co-alteration was the *TERT* C228T mutation and in the second case, the *TP53* K320* nonsense mutation. In one patient with multifocal PTC, the *ETV6/NTRK3* rearrangement was identified in a nodule in the right thyroid lobe and the *BRAF* V600E mutation in a nodule in the left thyroid lobe.

The carcinomas harboring *NTRK3* fusions (*n* = 49) were compared to the carcinomas with *NTRK1* fusions (*n* = 10) (Table 2). *NTRK3* fusion genes were almost 5 times more common than *NTRK1* fusions. The carcinomas positive for *NTRK1* fusions had a significantly more common mixture of papillary and follicular growth patterns (*p* = 0.006) than the carcinomas positive for *NTRK3* fusions, which had mostly predominant follicular growth patterns. Other differences were the significantly higher frequencies of multifocality (*p* = 0.015), extrathyroidal extension (*p* = 0.008), intravascular invasion (*p* = 0.024) and distant metastases (*p* = 0.013) of *NTRK1* fusion-positive carcinomas. The patients with carcinomas harboring *NTRK1* fusions had LN metastases 80% of the time compared to 49% of time for *NTRK3* fusion-positive carcinomas. However, the difference only approached the limit of statistical significance (*p* = 0.072).

Clinicopathological features of *NTRK* fusion-positive carcinomas were also compared between pediatric and adult patients (Supplementary Table S4). No statistically significant difference was observed between these cohorts.

### 3.3. Treatment and Follow-Up

The median follow-up was 63 months (range: 2–203 months). Fifty-one (86.4%) patients received radioactive iodine (RAI) treatment; eight patients (13.6%) received no RAI treatment due to microcarcinoma without aggressive features. The median cumulative dose of RAI per patient was 6.3 GBq (range: 1.9–28.0 GBq). Most patients (84.3%) were RAI responsive and accumulated RAI in thyroid remnants or in metastases. In addition to RAI therapy, both PDTC cases were also treated with external beam radiotherapy. The first patient with PDTC harboring two rare isoforms of *ETV6/NTRK3* fusions discontinued radiation treatment due to intolerance and subsequently died. The second patient with PDTC harboring *SQSTM1/NTRK1* was still alive (12 months after surgery). One patient with PTC and other two malignancies (sigmoid colon cancer diagnosed before TC and breast cancer diagnosed after TC), harboring *TPM3/NTRK1* and *TERT* C228T mutations in *PTC*, underwent chemotherapy treatment and subsequently died.

**Figure 2:**
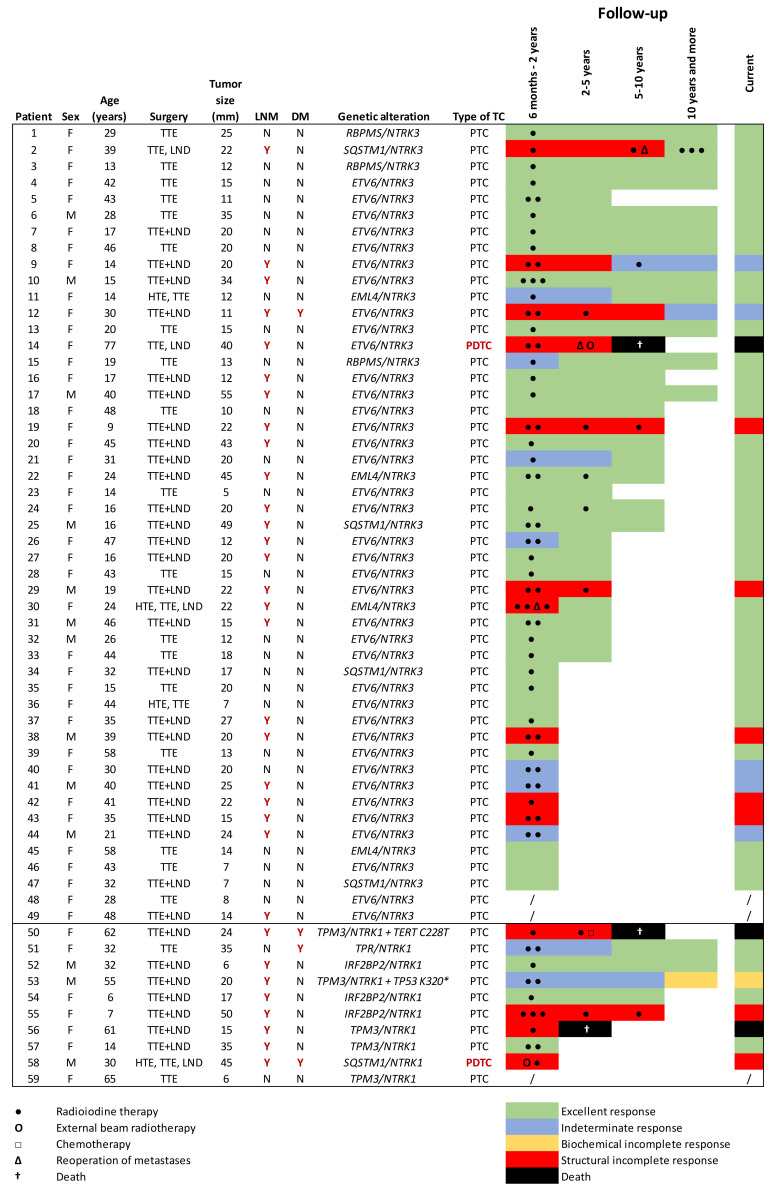
Detailed follow-ups and the outcomes of patients harboring *NTRK* fusion-positive carcinomas. F, female; M, male; the, hemithyroidectomy; TTE, total thyroidectomy; LND, lymph node dissection; LNM, lymph node metastases; DM, distant metastases; TC, thyroid cancer; Y, yes; N, no; PTC, papillary thyroid carcinoma; PDTC, poorly differentiated thyroid carcinoma.

Each responses to treatment was determined using definitions and criteria in the 2015 American Thyroid Association Guidelines as an excellent response (ER), an indeterminate response (IR), a biochemical incomplete response (BIR), a structural incomplete response (SIR), [18] or death. The response to treatment was assessed in the following time periods after surgery: 6 months to 2 years, 2–5 years, 5–10 years, 10 years or more and current response. Detailed follow-ups describing the courses of treatment in individual patients are displayed in Figure 2. Follow-up data of one patient were unknown and two patients were not evaluated due to short-term follow-up (less than six months after surgery).

**Figure 3:**
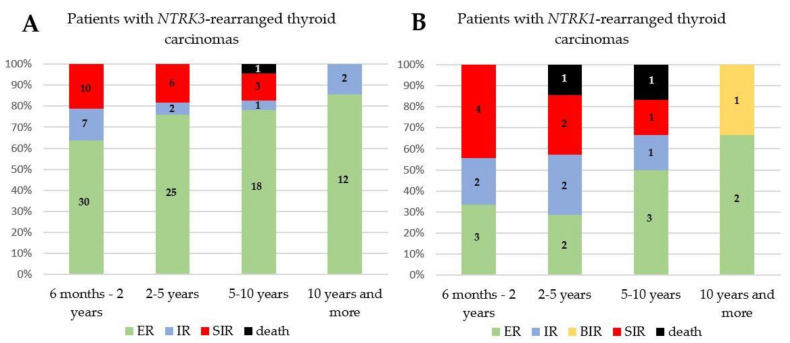
A graphical representation of the responses to treatment of the patients harboring *NTRK3* (**A**) and *NTRK1* (**B**) fusion-positive carcinomas after surgery. ER, excellent response; IR, indeterminate response; BIR, biochemical incomplete response; SIR, structural incomplete response.

Initially, during two years of treatment, 58.9% of *NTRK* fusion-positive patients had ER, 16.1% had IR and 25.0% had SIR. When patients were grouped according to the *NTRK3* or *NTRK1* mutation, 63.8% of *NTRK3* fusion-positive patients had ER and 21.3% had SIR. In the case of *NTRK1* mutation, more patients had SIR (44.4%) than ER (33.3%). During follow-up, three patients from the cohort who until then had SIR, died. One patient succumbed to PDTC; another two PTC patients were polymorbid. In *NTRK* fusion-positive cohort, the 2, 5 and 10-year overall survival rates were 100.0% (40/40), 96.7% (29/30) and 85.0% (17/20), respectively. However, during follow-up, response to treatment had an improving tendency in *NTRK3* as well as in *NTRK1* fusion-positive patients (Figure 3). After 10 years of follow-up, 82.3% of patients had ER, 11.8% had IR, 5.9% had BIR and no patient had an SIR. No case of recurrence was reported during follow-up when the ER to the patient’s treatment changed to BIR, SIR, IR or death. The patients without metastases had predominantly ER, and 16.7% of them had IR. Only one patient from the cohort who had SIR improved to ER. The reason for the improvement of the patient’s condition was the resection of LN metastases seven years after the initial surgery and subsequent RAI therapy. 

The prognosis of the disease also depended on the size of the tumor. All patients with a microcarcinoma (≤10 mm) had ER to treatment. However, it should be mentioned that one patient with an 11 mm carcinoma had metastases in 25 LN and also in the lungs. After 12 years of follow-up, the patient still had a high level of thyroglobulin antibodies. The patients with the tumor size ≤20 mm (*n* = 38) had LN metastases 36.8% of the time; and during the initial 2 years of treatment, 68.6% had ER, 17.1% had IR and 14.3% had SIR. The patients with tumors larger than 20 mm (*n* = 21) had LN metastases 85.7% of the time; and during the initial 2 years of treatment, 42.9% had an ER, 14.3% had IR and 42.9% had SIR. Both patients with PDTC had a tumor larger than 40 mm. 

## 4. Discussion

Molecular testing of genetic alterations in TC can be beneficial in several ways. It can help to establish preoperative diagnosis, the extent of surgery that is possible, estimate prognosis and determine appropriate treatment. Molecular markers, such as the *BRAF* V600E mutation or the *TERT* mutation, have been well studied in large cohorts, and the management of thyroid nodules harboring these mutations has also been recommended in the American and the European Thyroid Association Guidelines [18,19]. However, data on *NTRK* fusion genes in TC are very limited due to their rare occurrence, and therefore there are only small cohorts of *NTRK* fusion-positive samples that are described and characterized [10,11,12,15,20,21,22,23,24]. Another reason is their more expensive and challenging accurate testing requiring RNA of suitable quality. 

In this study, we retrospectively screened a large cohort of different thyroid tissue samples, including PTCs, PDTCs, ATCs, HCCs, FTCs, sporadic MTCs, NIFTPs, FT-UMPs and benign thyroid tissues, for *NTRK* fusion genes. *NTRK* fusion genes were detected in 59 TC samples, which to the best of our knowledge represents the largest cohort of *NTRK* fusion-positive samples in TC worldwide. The prevalence was 6.0% in all tested types of TC and 6.7% in PTCs, which was slightly higher than the prevalence around of 3% reported in PTCs in the literature [8,10,11,12]. One of the reasons for this result was a higher proportion of pediatric PTCs (*n* = 104) in our cohort, which are known to harbor *NTRK* fusions with a higher frequency (around 20%) [13,14,15]. After dividing the subjects into pediatric and adult patients, the prevalences of *NTRK* fusion genes in PTCs were 16.3% and 5.7%, respectively. No *NTRK* fusion gene was detected in any benign tissue sample (benign tumors, CLT). Thus, all eight tested types of *NTRK* fusions seem to be associated with a 100% probability of malignancy.

The low detection rate of *NTRK* fusions in adult PTC samples could discourage routine testing. However, as reported in another study, after elimination of samples harboring the *BRAF* V600E mutation, the detection rate of *NTRK* fusion genes increased up to 20% [11]. *NTRK* fusions are typically mutually exclusive with the *BRAF*, *HRAS*, *KRAS*, *NRAS* and other driver mutations [8,10,12,13,22]. Therefore, in our study, the samples positive for mutations in the *BRAF*, *HRAS*, *KRAS* and *NRAS* genes, and for *RET/PTC* or *PAX8/PPARγ* were excluded from the *NTRK* fusion gene analyses, and then the detection rate of *NTRK* fusions was 22.8%. The co-occurrence of *NTRK* fusions with the *BRAF* V600E mutation has been described, but is very rare [21,23]. In contrast, the co-occurrence with late mutation events such as *TERT* or *TP53* mutations has been reported in several studies [9,25,26] and was also confirmed in this study. In one multifocal PTC in our study, the *NTRK* fusion gene was found in one thyroid nodule and the *BRAF* V600E mutation in another. Thus, it is important to point out the need to test all nodules in a case of multifocal TC due to the possibility of detection of different mutations, which could affect the patient’s treatment. 

All eight identified *NTRK* fusion types have been previously described and are recurrent in TC [27]. One rare isoform, *ETV6*(exon 2)/*NTRK3*(exon 14), detected in the PDTC sample, is unlikely to be oncogenic due to the lack of the SAM domain of ETV6 necessary for the fusion protein oligomerization. The SAM domain is encoded by exons 3 and 4 of the *ETV6* gene. The same PDTC sample also harbored another isoform, *ETV6*(exon 5)/*NTRK3*(exon 15), whose exons 3 and 4 of the *ETV6* gene were preserved. Only *NTRK1* and *NTRK3* fusions were identified similarly to other studies [11,22,25]. *NTRK2* fusions generally occur in other types of cancer, especially in gliomas. However, one case of the *NTRK2* fusion (*CRNDE/NTRK2*) in ATC has been described in the literature [28].

In this study, we performed a detailed clinicopathological review to investigate characteristic features of *NTRK* fusion-positive tumors. *NTRK* fusions were associated with the follicular growth pattern, as was also observed previously [11,14,20,21,25]. The predominant follicular pattern was more common in *NTRK3* than in *NTRK1* fusion-positive carcinomas, in which the follicular architecture mostly co-occurred with foci of papillary formation. Additionally, in most (65.5%) patients with *NTRK* fusion-positive carcinomas, the CLT was identified. The same findings were concluded in other studies, in which, surprisingly, similar frequencies of CLT were found (66.7% and 69.2%) [20,22]. 

Regarding the aggressiveness of *NTRK* fusion-positive carcinomas, data from the publications differed. Chu et al. suggested that *NTRK* fusion-positive carcinomas are associated with clinically aggressive disease with a high metastatic rate. In their first publication including ten *NTRK* fusion-positive PTCs, the mean tumor size was 54.2 ± 23.0 mm; 100% of patients had lymphovascular invasion and LN metastases; and 60% of patients had distant metastases [25]. Their following publication included additional *NTRK* fusion-positive patients with a total of 18 patients. Extrathyroidal extension and LN metastases were then identified in 61.1% and 77.8% of patients, respectively [27]. Fazeli et al. identified distant metastases in 77% of PTC/PDTC patients [23]. All these studies were biased due to case selection, in the case of Chu et al., the authors selected cases based on unusual morphology and advanced presentation. Fazeli et al. chose patients with advanced or RAI refractory cancer [23,27]. 

In our study, there was no case selection based on the aggressivity of disease. The mean tumor size was 20.9 ± 11.9 and extrathyroidal extension was identified in 40.7%, multifocality in 44.1%, vascular invasion in 23.5%, LN metastases in 54.2% and distant metastases in 6.8%. These findings are in accordance with data found in studies of small cohorts, in which also no selection based on aggressivity of disease was applied: mean tumor size 19.1–33.0 mm [11,12,15,20,22], extrathyroidal invasion in 0–33.3% [11,15,20,22], multifocality in 23.1–66.7% [15,20,22], vascular invasion 16.7–71.4% [15,20], LN metastases in 41.7–72.7% [11,12,15,20,22] and distant metastases in 8% [11,20]. 

Furthermore, based on our data, *NTRK1* fusion-positive carcinomas were significantly associated with a higher frequency of tumor multifocality and distant metastases, and were more invasive than the carcinomas harboring a *NTRK3* fusion. There are no records of comparison between *NTRK1* and *NTRK3* fusion-positive carcinomas in the literature. In addition, in two of our *NTRK1* fusion-positive cases, we identified a late mutation event (*TERT* and *TP53* mutation), known to correlate with progressive disease behavior [29]. In contrast, in the literature, late mutation events were detected more frequently along with *NTRK3* fusions than with *NTRK1* fusions, in four and two cases, respectively [9,26,27,30]. 

In this study, we identified two cases of PDTC harboring a *NTRK* fusion gene. Both carcinomas also had a PTC component of a mixed papillary and follicular growth pattern. Thus, we hypothesize that both PTCs harboring a *NTRK* fusion dedifferentiated to PDTCs. In both PDTCs, only *NTRK* fusion was detected without further mutations, so we assume that it was a *NTRK* fusion-driven progression. This finding was also supported by two studies, in which PDTCs with a PTC component were observed [28,31]. The same feature for both PDTCs was the tumor size larger than 40 mm, indicating the importance of early surgery due to a risk of dedifferentiation of large carcinomas harboring a *NTRK* fusion gene. 

Tumor size was also one of the factors affecting the outcome that were identified during follow-up of patients. We demonstrated that the size of the *NTRK* fusion-positive carcinoma was directly related to the frequency of metastases. The patients with the tumor size ≤20 mm had LN metastases in 36.8% of the time, and those with a tumor larger than 20 mm had LN metastases 85.7% of the time. All patients with microcarcinomas had excellent prognosis and almost all patients without metastases achieved a favorable outcome. On the other hand, more than half of patients with metastases suffered from persistent disease and three patients died. Other indicators of worse outcome were *TERT* or *TP53* co-mutations and the presence of *NTRK1* fusion. However, it seems that recurrence risk is very low. No case was observed in which the patient had structural disease after remission. It is important to note that most patients underwent relatively radical surgery and received RAI treatment.

Follow-up data of patients with *NTRK* fusion-positive TC are very scarce in the literature. Two studies reported that most patients had no evidence of disease [11,20]. Different data were observed in a study biased with case selection based on the aggressivity of disease. Chu et al. reported that from 11 patients with evaluable follow-up, only two patients (18.2%) achieved remission, and the remaining nine patients (81.8%) had recurrence or persistence of the disease. Interestingly, both patients with no evidence of disease had a tumor of the smallest size (21 and 35 mm) from the cohort, harbored a *NTRK3* fusion gene and lacked any late mutation event [27].

Carcinomas of most patients in our study were RAI-avid. Therefore, RAI treatment seems to be a “gold standard” therapy for most *NTRK* fusion-positive carcinomas. In our study, to none of the patients had a TRK inhibitor been administrated. However, in the case of advanced or RAI-refractory carcinomas, patients may benefit from this kind of treatment. For the TRK inhibitor larotrectinib, a response rate of more than 75% was observed regardless of cancer type and was well tolerated by patients [32].

Due to the high risk of multifocality and 100% probability of malignancy, total thyroidectomy may be recommended as a minimal surgical procedure in the case of preoperative detection of the oncogenic *NTRK* fusion gene in the thyroid nodule. Cervical ultrasonography with LN mapping of central and lateral compartments should be preoperatively performed. In the case of a nodule larger than 20 mm or the presence of late mutation event (*TERT* or *TP53* mutation), prophylactic central LN dissection should be considered.

This study had a few limitations. First, as already mentioned, no patient received TRK inhibitor therapy. Second, 133 samples were only analyzed by real-time PCR, so that novel *NTRK* fusion genes could not be identified in these samples. 

## 5. Conclusions

In conclusion, we detected the *NTRK* fusion gene in 59 (6.0%) TC samples. These samples were associated with the follicular growth pattern of TC, chronic lymphocytic thyroiditis and a high rate of LN metastases, especially in larger carcinomas. Furthermore, *NTRK1* fusion-positive carcinomas differed from carcinomas harboring the *NTRK3* fusion gene by higher frequencies of tumor multifocality, distant metastases and carcinoma invasiveness. Additionally, we reported that the size of the tumor, positivity for *NTRK3* or *NTRK1* fusion, the presence of metastases and late mutation events may provide prognostic information for patient outcomes. Based on all these findings, testing of *NTRK* fusions could help to establish diagnosis, advise on extent of surgery, specify prognosis and set up personalized treatments.

## Figures and Tables

**Table 1:** Clinicopathological features of *NTRK* fusion-positive carcinomas.

	*NTRK* Fusion-Positive Carcinomas (%) *n* = 59
**Patients**	
Females/males	46/13
Age at diagnosis (mean ± SD)	32.8 ± 16.0
History of radiation exposure before the diagnosis of TC	1 (1.7)
History of prior malignancy before the diagnosis of TC	1 (1.7)
**Tumor size**	
Mean ± SD (mm)	20.9 ± 11.9
Microcarcinoma (≤10 mm)	8 (13.6)
**Histology**	
Predominantly papillary growth pattern	8 (13.6)
Mixture of papillary and follicular growth pattern	18 (30.5)
Predominantly follicular growth pattern	28 (47.5)
Tall cell variant	2 (3.4)
Clear cell variant	1 (1.7)
PDTC	2 (3.4)
**Pathological characteristics**	
Multifocality	26 (44.1)
Extrathyroidal extension	24 (40.7)
Intravascular invasion ^1^	12 (23.5)
Lymph node metastases	32 (54.2)
Distant metastases	4 (6.8)
Chronic lymphocytic thyroiditis ^2^	38 (65.5)
Frequent psammoma bodies ^3^	6 (10.3)

^1^ Data from eight samples were not available. ^2,3^ Data from one sample were not available. SD standard deviation, TC thyroid cancer, PDTC poorly differentiated thyroid carcinoma.

**Table 2:** Comparison of clinical and pathological features between *NTRK3* and *NTRK1* fusion-positive carcinomas.

	*NTRK3* Fusion-Positive Carcinomas (%) *n* = 49	*NTRK1* Fusion-Positive Carcinomas (%) *n* = 10	*p*
**Patients**			
Females/males	39/10	7/3	0.383
Age at diagnosis (mean ± SD)	32.0 ± 14.4	36.4 ± 21.9	0.426
History of radiation exposure before the diagnosis of TC	0	1 (10.0)	0.169
History of prior malignancy before the diagnosis of TC	0	1 (10.0)	0.169
**Tumor size**			
Mean ± SD (mm)	20.0 ± 11.0	25.3 ± 14.6	0.195
Microcarcinoma (≤10 mm)	6 (12.2)	2 (20.0)	0.409
**Histology**			
Predominantly papillary growth pattern	8 (16.3)	0	0.203
Mixture of papillary and follicular growth pattern	11 (22.4)	7 (70.0)	**0.006**
Predominantly follicular growth pattern	26 (53.1)	2 (20.0)	0.057
Tall cell variant	2 (4.1)	0	1
Clear cell variant	1 (2.0)	0	1
PDTC	1 (2.0)	1 (10.0)	0.313
**Pathological characteristics**			
Multifocality	18 (36.7)	8 (80.0)	**0.015**
Extrathyroidal extension	16 (32.7)	8 (80.0)	**0.008**
Intravascular invasion ^1^	7 (16.7)	5 (55.6)	**0.024**
Lymph node metastases	24 (49.0)	8 (80.0)	0.072
Distant metastases	1 (2.0)	3 (30.0)	**0.013**
Chronic lymphocytic thyroiditis ^2^	34 (70.8)	4 (40.0)	0.069
Frequent psammoma bodies ^3^	4 (8.3)	2 (20.0)	0.274

Values highlighted in bold were statistically significant. ^1^ Data from seven NTRK3 fusion-positive samples and from one NTRK1 fusion-positive sample were not available. ^2,3^ Data from one NTRK3 fusion-positive sample were not available. SD standard deviation, TC thyroid cancer, PDTC poorly differentiated thyroid carcinoma.

## Data Availability

The data presented in this study are available on reasonable request from corresponding author.

## Supplementary Materials

The following are available online at https://www.mdpi.com/article/10.3390/cancers13081932/s1. Supplementary Table S1: Sequences of primers and hydrolysis probes designed for the *ACTB* gene and the detection of *NTRK* fusion genes using real-time PCR. Supplementary Table S2: Comprehensive clinicopathological data on *NTRK* fusion-positive carcinomas. Supplementary Table S3: Transcripts of genes involved in *NTRK* rearrangements Supplementary Table S4: Comparison of clinical and pathological features between pediatric and adult *NTRK* fusion-positive carcinomas.
